# Competitive Salt Precipitation/Dissolution During Free‐Water Reduction in Water‐in‐Salt Electrolyte

**DOI:** 10.1002/anie.202005378

**Published:** 2020-06-22

**Authors:** Roza Bouchal, Zhujie Li, Chandra Bongu, Steven Le Vot, Romain Berthelot, Benjamin Rotenberg, Frederic Favier, Stefan A. Freunberger, Mathieu Salanne, Olivier Fontaine

**Affiliations:** ^1^ ICGM Univ. Montpellier, CNRS Montpellier France; ^2^ Sorbonne University, vUPMC Univ. Paris 06 CNRS, Laboratoire PHENIX 75005 Paris France; ^3^ Maison de la Simulation CEA, University Paris-Saclay 91191 Gif-sur-Yvette France; ^4^ Institute for Chemistry and Technology of Materials Graz Stremayrgasse 9 8010 Graz Austria; ^5^ IST Austria (Institute of Science and Technology Austria) Am Campus 1 3400 Klosterneuburg Austria; ^6^ Réseau sur le Stockage Electrochimique de l'Energie (RS_2_E) CNRS FR3459 33 rue Saint Leu 80039 Amiens Cedex France

**Keywords:** electrochemistry, electrolytes, interfaces, salt effect, water-in-salt

## Abstract

Water‐in‐salt electrolytes based on highly concentrated bis(trifluoromethyl)sulfonimide (TFSI) promise aqueous electrolytes with stabilities nearing 3 V. However, especially with an electrode approaching the cathodic (reductive) stability, cycling stability is insufficient. While stability critically relies on a solid electrolyte interphase (SEI), the mechanism behind the cathodic stability limit remains unclear. Now, two distinct reduction potentials are revealed for the chemical environments of free and bound water and that both contribute to SEI formation. Free water is reduced about 1 V above bound water in a hydrogen evolution reaction (HER) and is responsible for SEI formation via reactive intermediates of the HER; concurrent LiTFSI precipitation/dissolution establishes a dynamic interface. The free‐water population emerges, therefore, as the handle to extend the cathodic limit of aqueous electrolytes and the battery cycling stability.

## Graphical abstract

**Water‐in‐salt** electrolytes have received particular attention for their electrochemical stability properties. It is now shown that water reduction is always present from the first measured currents, and that associated with this water reduction, LiTFSI precipitation occurs.

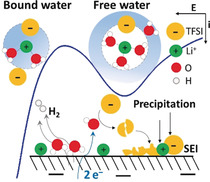

Water‐in‐salt (WIS) electrolytes have recently emerged as new promising electrolytes owing to their high electrochemical stability window (ESW) of about 3 V.^1^, ^2^ The drastic widening of the ESW could increase the energy of an electrochemical cell fourfold compared to conventional aqueous electrolytes, making WIS attractive, safe alternatives to hazardous organic electrolytes.^3^, ^4^, ^5^ However, poor cycling stability hinders practical application for the critical case of low‐voltage negative electrodes that operate below about 1.9 V vs. Li/Li^+^.^6^, ^7^, ^8^ ESWs reported to date with sufficient cycling stability are hence far below the expected 3 V.^2^, ^5^, ^9^, ^10^, ^11^, ^12^

Ever since the promise of WIS electrolytes has been realized, extensive efforts have been devoted to understanding the origin of ESW stabilization. The high salt concentration in WIS significantly changes the liquid structure and alters interfacial reactivity.^2^, ^4^ At the positive electrode, it is widely agreed that anions accumulate at the interface, forming a dense hydrophobic layer that prevents contact between water and electrode,^13^, ^14^, ^15^ which raises the onset of oxygen evolution from 3.8 V (at pH 7) to about 4.9 V vs. Li/Li^+^.^1^, ^2^, ^6^, ^16^, ^17^ At the negative side, most studies assigned the improved cathodic stability to SEI formation from TFSI reduction,^4^, ^14^ which hinders water molecules to access the surface while allowing for Li^+^ diffusion.^2^, ^8^, ^14^ The SEI kinetically suppresses the hydrogen evolution reaction (HER) and shifts the cathodic limit from 2.6 V (at pH 7) to about 1.8 V vs. Li/Li^+^.^2^, ^8^ Since the SEI forms only beyond salt concentrations at 21 m (molal, mol per kg solvent), it was argued to arise from bound water molecules having a lower reduction potential than TFSI.^7^, ^8^, ^9^ However, the assignment of the reduction wave to either HER or TFSI reduction is unclear and several studies have reported on a rather wide range of potentials for TFSI reduction between 2 and 2.5 V vs. Li/Li^+^.^8^, ^14^, ^18^ Two conflicting explanations were recently brought forward by Suo et al.^14^ and Dubouis et al.^19^ The first reported the competitive reduction of water, dissolved O_2_, CO_2_, and TFSI during formation of an SEI comprising Li_2_CO_3_ and LiF. They concluded that the TFSI in the ionic clusters and water are reduced at the same potential, where H_2_ evolution is considered as a parasitic reaction for the SEI formation. Dubouis et al.^19^ found simultaneous decomposition of water and TFSI with TFSI being chemically decomposed by products from the HER and not electrochemically as suggested previously.

These conflicting explanations raise questions about what truly causes the enhanced reductive stability and what nevertheless limits it. If direct TFSI reduction forms the SEI, then it is unclear why the TFSI would in the aqueous environment be reduced at much higher voltage (2 to 2.5 V vs. Li/Li^+^) than in organic electrolytes (0.5–1.5 V vs. Li/Li^+^).^20^, ^21^, ^22^ If HER is the only responsible for the SEI formation, then TFSI decomposition should occur at all salt concentrations. Taken together, it is not clear what truly determines the reductive stability of WIS electrolytes and what the processes upon reduction are. Increased stability may be caused by passivation, the unusual state of water or the bulk properties, such as viscosity and ion‐pair and anion‐cation aggregates, and, if it is caused by the passivation layer, it is not clear how it forms. Only a better understanding of the electrochemical reduction of water in WIS electrolytes, together with the reactions during SEI formation, would allow the understanding of the stability enhancement, its limitations, and handles to improve further reductive stability, cycling stability, and energy density.

Herein, we combine electrochemistry and spectroscopy with molecular dynamics (MD) calculations to understand the processes at the interface during SEI formation. Quantitative online electrochemical mass spectrometry shows that water reduction is the only contributor to the reductive current. Since the electrolyte is nearly saturated, water reduction causes local oversaturation at the electrode interface. Salt precipitation/dissolution, together with two distinct water reduction potentials, provide handles to understand/improve reductive stability.

Linear polarization experiments, performed in WIS with increasing concentrations on a rotating glassy carbon electrode, show very different behaviors on reduction and oxidation, as shown in Figure 1 a and the Supporting Information, Figures S1 and S2, respectively. Our results for enhanced oxidation are in accord with previous reports.^13^, ^14^, ^15^, ^17^ They show that high salt concentrations displace water with TFSI at the interface, adding a higher thermodynamic barrier for water to reach the electrode surface and to become oxidized. During negative polarization, the voltammograms show a much more complex pattern as the concentration rises (Figure 1 a). Below 12 m, there is one reduction with an onset potential of about 1.8 V as observed for dilute aqueous Li_2_SO_4_ solution. At 12 m and above, this process turns into a reduction wave with a distorted bell shape that peaks at about 1.2 V with nearly unaltered onset, thus suggesting that it corresponds to water reduction in all cases. Both the charge under the peak and the kinetics decrease with increasing salt concentration to a nearly vanishing yet still clearly visible magnitude at 20 m (Supporting Information, Figure S3). For linear polarization showing a peak (≥12 m), a second reduction occurs with an onset at about 0.7 V and equally decreasing and peaking magnitude. We hypothesize that there are two different water populations with different chemical environments, which are reduced at two different potentials (see the Supporting Information for more explanation).

**Figure 1:**
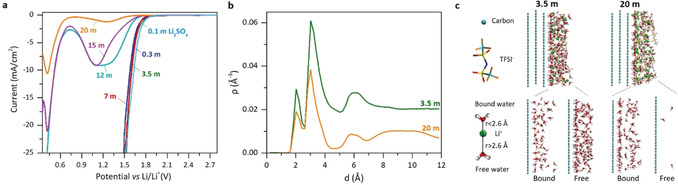
a) Linear polarization at a rotating glassy carbon disk electrode with different salt concentrations (molality) at 1000 min^−1^, a scan rate of 1 mV s^−1^, and 25 °C. Curves versus Ag/AgCl and Fc/Fc^+^ are given in the Supporting Information, Section S3. b) Water density profiles at the negative electrode from MD simulations with 3 V potential difference between graphite electrodes. c) Representative snapshots of the whole adsorbed layer (top), and only the bound and free water molecules (bottom).

The existence of two water populations is in accord with the water density profiles obtained from MD simulations (Figure 1 b,c; Supporting Information, Figure S4). They show 3.5 and 20 m electrolytes in contact with the negative graphite electrode with a potential difference of 3 V to another graphite electrode. The densities decrease across the whole interface as the water‐to‐salt ratio decreases when going from 3.5 m to 20 m, but the positions of the peaks remain the same. Although water molecules are in both cases in contact with the surface, their coordination states differ strongly, as the snapshots in Figure 1 c show. A large amount of free water at 3.5 m is opposed to nearly all water coordinating Li^+^ at 20 m (*d*_Li‐O_<2.6 Å, water molecule in the first lithium solvation shell), leaving only a minority uncoordinated. Whether or not water coordinates Li^+^ could hence change its reduction potential and cause the two experimentally observed potentials. Only minor free water implies the associated wave to have limited capacity.

The above results explain the reductive (cathodic) stability only based on water reduction; direct TFSI reduction to form a passivating layer as suggested in previous reports on Chevrel phase Mo_6_S_8_ electrodes^2^, ^14^ appears not to be involved. The results in the Supporting Information, Figure S6 confirm that water reduction, governed in the same way by the chemical environment of water molecules, passivates both carbon and Mo_6_S_8_ electrodes.

To quantitatively confirm our interpretation that the peaking reduction process at intermediate potentials is water reduction rather than TFSI reduction, we performed online electrochemical mass spectrometry (OEMS) on porous electrodes with 0.3 and 20 m electrolytes, as detailed in the Methods in the Supporting Information. While the working electrode was linearly polarized to reducing potentials, the cell head space was continuously purged to a mass spectrometer for gas analysis, and the results are presented in Figure 2 for the 20 m electrolyte and in the Supporting Information, Figure S8 for 0.3 m. For both salt concentrations, H_2_ is the only gas produced except for circa 1000‐fold lower CO (Supporting Information, Figure S9). The e^−^ and H_2_ fluxes are given in Figure 2, the cumulative moles in the Supporting Information, Figure S8. The e^−^/H_2_ ratio is very close to two as expected for the HER reaction H_2_O+2 e^−^→H_2_+OH^−^. Hence, the first reduction wave corresponds to water reduction and not to TFSI reduction.

**Figure 2:**
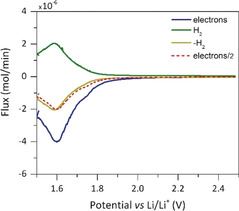
Online electrochemical mass spectrometry at reducing potentials with a porous carbon electrode at a scan rate of 0.1 mV s^−1^ in 20 m LiTFSI in H_2_O. Molar e^−^ and H_2_ fluxes as well as half the e^−^ flux.

Linear polarization and OEMS measurements show that the reaction limiting cathodic stability of the WIS electrolyte is the HER, which occurs at two different potentials for free and bound water. The absence of direct electrochemical TFSI reduction raises questions about the true SEI formation pathway for which two different and contradicting theories have been reported, as mentioned above.^14^, ^19^ In both cases, water is sacrificed during the SEI formation; this means that water will be continuously consumed upon cycling, leading to dryness and cell failure.

To understand how water reduction can affect the TFSI precipitation, we set up a simple model to calculate the TFSI concentration profile at the interface (details in the Supporting Information and Figures S10, S11 therein). Note that the concentrations are apparent ones, since they may exceed solubility, which is about 5.1 m in the bulk liquid, corresponding to 22–23 m and 25 °C.^2^ The amount beyond this concentration hence precipitates. Figure 3 shows the evolution of the LiTFSI concentration at the interface during polarization with 7, 15, and 20 m electrolytes. LiTFSI precipitates independent of the molality at about 1.3 V–1.5 V vs. Li/Li^+^. At low concentration (7 m), the LiTFSI concentration keeps increasing with the water reduction in accord with the sharply rising current (Supporting Information, Figure S11a). This means that H_2_O is supplied again from the bulk at a rate balancing the reduction at the interface since viscosity is low. In contrast, for 15 and 20 m (where the current peaks around 1.2 V), the LiTFSI concentration only slightly exceeds saturation and levels off towards low oversaturation (see Figure 3 b and the Supporting Information, Figure S11b, respectively). This means that a solid LiTFSI layer is established, and water resupply is too low to dissolve once again the LiTFSI, likely by a combination of high viscosity, the LiTFSI layer, low H_2_O gradient, and near absence of free water in the bulk electrolyte. Of note, we do not exclude a certain Li^+^ conductivity beyond the value of dry LiTFSI^23^ in the precipitated LiTFSI since it will remain slightly hydrated. For high LiTFSI concentrations, the diffusion flux of water, Li^+^, and TFSI decreases (because the medium is more viscous, and the concentration gradient is lower). Therefore, water flow from the solution to the electrode and ions flow from the electrode to the solution are the driving forces for dissolution. The driving force of the precipitation will be the applied overvoltage, and the increase of the local viscosity. The precipitation/dissolution model helps to rationalize many phenomena in high‐concentration electrolytes. It explains the experimental observations that the SEI formed is said to be dynamic, that is, it tends not to stick to the electrode upon cycling.^8^, ^14^ If a significant portion of the SEI layer is precipitated salt, then the SEI is dynamic and decomposes/reconstructs during polarization.

**Figure 3:**
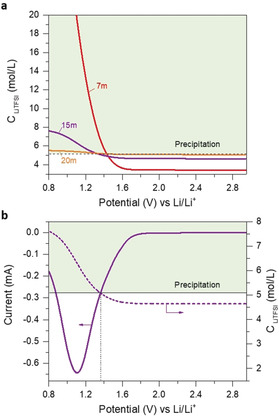
Salt concentration profile at the interface. a) Apparent C_LiTFSI_ profile with 7, 15, and 20 m electrolytes. b) C_LiTFSI_ profile and linear sweep voltammetry of the 15 m solution.

Our results show no electrochemical signature for TFSI reduction at free water reduction potential and confirm the results of Dubouis et al.,^19^ on the chemical decomposition of TFSI during HER process. We go beyond that discriminates precipitation from TFSI decomposition at reduction potentials of both water populations. To do so, we analyzed the surface of binder‐free porous carbon nanofiber polarized at 1.2 V and 0.4 V vs. Li/Li^+^, corresponding to the reduction of free water and bound water, respectively (experimental details and results for Mo_6_S_8_ electrodes are given in the Supporting Information).

SEM images of the CNF electrodes after polarization (Figure 4 a–c; Supporting Information, Figure S12) show some material deposition on the fibers at both potentials but larger amounts at lower potential. Energy‐dispersive X‐ray spectroscopy (EDS) clearly shows N, O, F, and S containing deposits at all electrode surfaces (Supporting Information, Figure S13). The polarized electrodes differ from the pristine sample is strongly increased O and F atom fractions at the expense of the C fraction, thus showing increasing carbon surface coverage with decreasing potential.

**Figure 4:**
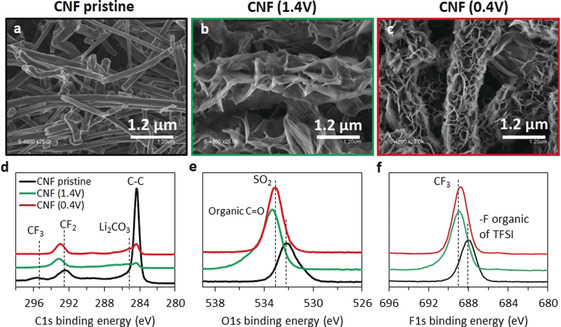
a)–c) SEM and X‐ray photoelectron spectroscopy (XPS) analysis of carbon nanofibers in 12 m WIS solution. (a–c), SEM figures of pristine CNF (a), CNF polarized to 1.4 V (b), and CNF polarized to 0.4 V (c) for 15 min, respectively. d)–f) (XPS) spectrum of CNF electrodes. Black, green, and red curves correspond to the pristine CNF, CNF(1.4 V), and CNF(0.4 V) electrodes, respectively.

X‐ray photoelectron spectroscopy (XPS) further identified the species in the deposits. Figure 4 e,f and the Supporting Information, Figure S14 show the C 1s, O 1s, F 1s, S 2p, N 1s, and Li 1s spectra of the CNF. The intensity of C−C peaks in the C 1s spectra strongly decreases for the polarized samples. The presence of ethereal carbon species (CO_1,2,3_) was observed at 286 eV, next to a new peak at 293 eV corresponding to C−F_2_. The latter is in accord with some decomposition of TFSI during polarization since the electrodes are free of any fluorinated binder. O 1s, F 1s, S 2p, and N 1s spectra show peak shifts toward higher energies after polarization, which all can be assigned to decomposition products of TFSI. An O 1s peak at 533.2 eV and S 2p peaks above 169 eV are characteristics of various −SO_2_ or −SO_3_ containing species. Fluorine species such as C−F_3_ are detected at 688.8 eV and nitrogen‐containing surface species (such as Li_2_NSO_2_CF_3_) at about 400 eV in the F 1s and N 1s spectra.^24^ Mo_6_S_8_ electrodes show mainly the same decomposition products as on CNFs at 1.4 V, with an additional peak characteristic for Li_2_CO_3_, which we assign mostly to the oxidation of the carbon counter electrode. Our interpretation is supported by the absence of Li_2_CO_3_ at the CNF electrode when the counter electrode was a platinum disk but its presence when it was equally CNFs (see the Supporting Information for details). Overall, carbon and Mo_6_S_8_ electrodes are covered with the same surface species when polarized to potentials that drive water reduction. Unlike the previously reported SEI in WIS electrolytes, we did not detect any LiF. This difference could be related to the potentiostatic polarization method in our experiment. Our results show that TFSI is chemically decomposed during free‐water reduction. However, at the bound water reduction, TFSI appears to be degraded both chemically and electrochemically.

By combining electrochemical and analytical techniques with MD simulations, we have shown that the only reactions limiting WIS electrolyte stability are the oxygen and hydrogen evolution reactions (OER and HER). We demonstrated that HER occurs at two different potentials, which correspond to the populations of free and bound water. The ESW is increased thermodynamically at the anodic side, while H_2_O reduction is negligibly at negative potentials; the handle is only the amounts in these populations. A precipitation/dissolution mechanism of LiTFSI at the electrode interface is brought up as a new process during the SEI formation. Together with the salt precipitation, we revealed that TFSI anions are chemically decomposed during water splitting even at a lower concentration than the previously reported 21 m.

The competition between salt precipitation and dissolution during H_2_O reduction renders the SEI dynamic and not stable, which builds and decomposes upon cycling. This mechanism could be generalized to more concentrated electrolytes that contain a limited proportion of solvent. A salt precipitation at the interface, governing the formation of SEI, is an assumption that should not be overlooked now.

## Conflict of interest

The authors declare no conflict of interest.

## Supporting information

As a service to our authors and readers, this journal provides supporting information supplied by the authors. Such materials are peer reviewed and may be re‐organized for online delivery, but are not copy‐edited or typeset. Technical support issues arising from supporting information (other than missing files) should be addressed to the authors.

Supplementary
