# Supplementary material for: Competitive Salt Precipitation/Dissolution During Free‐Water Reduction in Water‐in‐Salt Electrolyte
Source: Angew Chem Int Ed Engl. 2020 Jun 22;59(37):15913–7. doi: 10.1002/anie.202005378 (PMC7540070; doi:10.1002/anie.202005378)
Supplement: Supplementary file 1 — Supplementary [file ANIE-59-15913-s001.pdf]

Angewandte Chemie
Eine Zeitschrift der Gesellschaft Deutscher Chemiker

Supporting Information

Competitive Salt Precipitation/Dissolution During Free-Water Reduction in Water-in-Salt Electrolyte

Roza Bouchal, Zhujie Li, Chandra Bongu, Steven Le Vot, Romain Berthelot, Benjamin Rotenberg, Frederic Favier, Stefan A. Freunberger, Mathieu Salanne, and Olivier Fontaine**

anie_202005378_sm_miscellaneous_information.pdf

Author Contributions

R.B. Investigation: Equal; Methodology: Equal; Writing—Original Draft: Equal
Z.L. Data curation: Equal; Software: Supporting
C.B. Data curation: Supporting
S.L. Methodology: Supporting
R.B. Resources: Supporting
B.R. Formal analysis: Equal; Validation: Equal; Visualization: Equal
F.F. Methodology: Supporting; Supervision: Supporting
S.F. Conceptualization: Supporting; Data curation: Supporting; Formal analysis: Supporting; Funding acquisition: Supporting; Methodology: Equal; Resources: Supporting; Visualization: Supporting; Writing—Original Draft: Supporting; Writing—Review & Editing: Equal
M.S. Formal analysis: Equal; Software: Lead; Validation: Equal; Writing—Review & Editing: Supporting
O.F. Conceptualization: Lead; Visualization: Lead; Writing—Original Draft: Lead; Writing—Review & Editing: Lead.

SUPPORTING INFORMATION

Table of contents

Experimental Procedures 2
Materials 2
Electrolyte preparation 2
Electrode fabrication 2
Electrode preparation for surface analysis 2
Characterization 2
Linear polarization characterization 3
Differential electrochemical mass spectrometry (DEMS) 3
MD simulations 3
Results and Discussion 3
Cathodic stability 3
Anodic stability 4
Water reduction at Mo_6S_8 electrodes 6
Precipitation/dissolution mechanism at the interface 7
Interface formation at Mo_6S_8 10
Supporting References 12

Experimental Procedures

Materials

Lithium bis(trifluoromethylsulfonyl)imide, 99% (LiTFSI) was purchased from IOLITEC. The salt was stoked in an argon glove box; LiTFSI was dried beforehand at 125 °C under vacuum during 48 hours. Ferrocene methanol was purchased from Alfa Aesar Deionized water with a resistivity of 18.2 MΩcm was used in all experiments.
Activated carbon (AC) PICA was purchased from JACOBY Industry. Carbon black (CB) (Acetylene Black, 100% compressed) from Alfa Aesar. Polytetrafluoroethylene (PTFE) (60 wt% in solution) and carbon nanofibers were purchased from Sigma Aldrich.

Electrolyte preparation

The electrolyte solutions were prepared by dissolving salt in the corresponding quantity of water. Due to the hygroscopic character of the salt, in order to minimize calculation errors, the weighing was carried out under argon atmosphere. These solutions were prepared by molality (moles number of salt in 1 kg of water).

Electrode fabrication

Pica based electrodes: AC, CB and PTFE were mixed in a 75:15:10 weight ratio. AC was used as active material, CB as electronic conductor additive and PTFE as binder. After adding acetone, the solution was stirred and heated at 55°C until solvent evaporation. The resulting paste was kneaded and spread until resulting in a homogeneous film with a thickness of about 150 µm.
Carbone nanofiber based electrode: a quantity of carbon nanofibers were dispersed in ethanol during 1 hour by stirring and sonication. The suspension was then filtered and the filtrate was pressed several times to obtain a self-standing layer.
Chevrel phase Mo_6S_8 based electrode: the electrodes composition was prepared according to K. Xu *et al*., Mo_6S_8, CB and PTFE were mixed in a 80:10:10 weight ratio. The mixture was pressed in a thin film and punched out in 6 mm diameter electrodes.

Electrode preparation for surface analysis

The electrodes were first polarized in 12 m electrolyte to various reducing potentials for 15 min to form a surface film, and the pristine electrode was immersed overnight in 12m solution. The electrodes were then recovered and rinsed intensively with water to remove excess electrolyte or precipitated salt, then dried at 80 °C for 24 h. Electrodes were polarized to 1.2 V and 0.4 V vs. Li/Li^+, corresponding to the reduction of free water and bound water, respectively.

Characterization

All the electrochemical analysis were recorded using VMP-300 Multi Potentiostat - Bio-Logic instrument. Scanning electron spectroscopy (SEM) and energy dispersive spectroscopy (EDS) analysis were recorded with an FEI scanning electron microscope. The X-ray photoelectron spectroscopy (XPS) analyzes are performed with the Thermo Electron ESCALAB 250 instrument. The excitation source is the monochromatic source, line Al Kα (1486.6 eV). The analyzed surface has a diameter of 400 µm. The photoelectron spectra are calibrated as binding energy with respect to the energy of the C = C component of the C1s carbon at 284.4 eV. X-ray diffraction recorded on PANalytical X'Pert in Bragg-Brentano configuration with CuKα radiation.

Linear polarization characterization

Rotating disk electrode: linear polarization was recorded in a three electrodes cell on a glassy carbon working electrode at different LiTFSI·H_2O concentrations, platinum was used as counter electrode and Ag/AgCl as a reference. The scan rate was 1mV/s and before all measurements. Before all measurements, the working electrode was cleaned by mechanical polishing and the solutions were degassed for 10 min under nitrogen. The ohmic drop is compensated. Same experiments were performed using an internal reference, 2mM of ferrocene methanol was introduced in different salt concentration.

Carbon nanofiber: linear polarization was recorded in a three electrodes Swagelok cell at 5mV/s scan rate. Freestanding carbon nanofiber as working electrode, platinum disk as a counter electrode and Ag/AgCl disk as a reference.

Chevrel phase Mo_6S_8 based electrode: linear polarization was recorded in a three electrodes Swagelok cell at 5mV/s scan rate. Freestanding Mo_6S_8 based electrodes as working electrode, $LiCoO_2$ based electrode as a counter electrode and Ag/AgCl disk as a reference.

Differential electrochemical mass spectrometry (DEMS)

The gases evolved upon polarization, were studied using differential electrochemical mass spectrometry coupled to linear polarization. In cell DEMS we used completely delithiated LFP as a counter electrode, activated carbon (PICA) based material as the working electrode and the partially delithiated LFP as a reference. We chose activated carbon due to the high specific area, which increases the reactivity of the electrode with the electrolyte. Several counter electrodes were used in order to avoid its polarization out of the stable potential window. We did the measurements with two different concentrations of LiTFSI.H_2O electrolyte: 0.3 molal and 20 molal corresponding to salt-in-water and water-in-salt solutions respectively.

MD simulations

We use molecular dynamics to simulate two cells consisting of 3.5 and 20 m LiTFSI, respectively, placed between two graphite electrodes. The first simulation box (3.5 m LiTFSI) contains 116 LiTFSI ion pairs and 1839 water molecules as the electrolyte, the second one (20 m LiTFSI) contains 255 LiTFSI ion pairs and 707 water molecules as the electrolyte, and both of them contains 2496 carbon atoms as the two graphite electrodes. We exploit the atomistic model with modified OPLS force filed for ions and the SPC/E model for water,[1,2] which has been validated as in our previous work.[3] The electrodes consist of three fixed graphene layers on each side with in-plane dimensions of L_x = 32.25 Å and L_y = 34.37 Å. The length of the cell in z-direction was set to L_x = 90 and 96 Å (for 3.5 m and 20 m, respectively) to match the experimental density of the electrolyte in the bulk region of the simulation cells. A vacuum region was added in the z-direction, and the Yeh−Berkowitz condition for slab correction was used to mimic 2D periodic boundary condition.[4]

The simulations were conducted in the NVT ensemble with a time step of 1 fs at room temperature (298.15 K) by using LAMMPS code.[5] The systems were first equilibrated during 80 ns at zero constant charge on each electrode and followed by 12 ns equilibration at zero constant potential. During the constant potential runs, the carbon atom charges on the electrode were allowed to fluctuate, which ensured an adequate description of the surface polarization by ions and water during simulations. The simulation data were finally collected in 8 and 25 ns production runs (for 3.5 and 20 m, respectively) at constant potential with configurations saved every 1 ps for each simulation.

Results and Discussion

Cathodic stability

With the used rotating disk electrode, the diffusion layer is in a stationary state as shown in Fig. S5. Its electrochemical response generally gives a current plateau or sharply rising current for low/high reactant concentration, respectively. The latter is seen for up to 7 m in Fig. 1a and Fig S1. At higher salt / lower free water concentrations, no plateau is seen, but a bell shaped peak. This is explicable by either the reduction of a finite amount of water in the diffusion layer and/or electrode blocking as a result of the water reduction. This resembles a thin-layer type behaviour, in which the electro-active molecules are confined in a finite volume.[6,7] The first reduction wave may therefore be associated to the reduction of the population of water which is a minority in the diffusion layer, i.e. the free water molecules, as shown by the simulation results in Fig.1c. Further, it means that this population is the less replenished from the bulk electrolyte reservoir the higher the salt concentration.

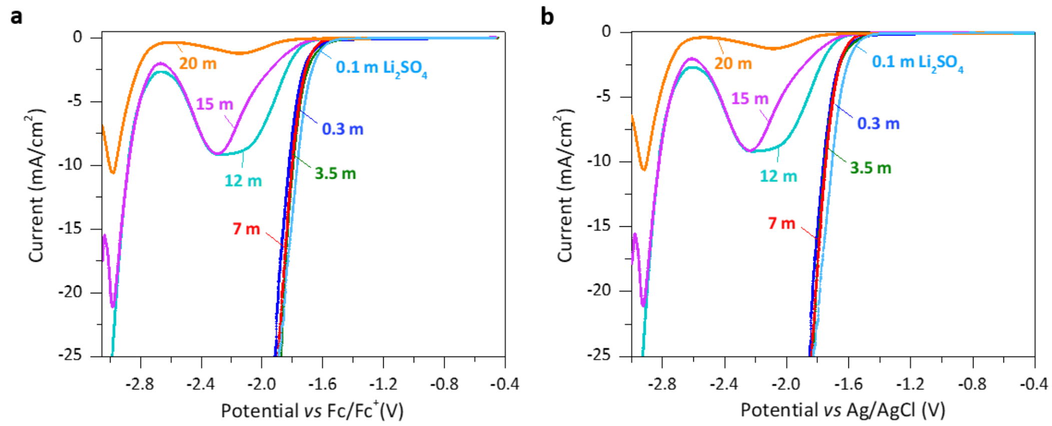

Figure S1. Electrochemical stability of LiTFSI-H_2O water-in-salt electrolytes. Linear polarization on a glassy carbon rotating disk electrode with different salt concentrations (molality). Platinum was used as counter electrode and Ag/AgCl as reference electrode. Curves were measured at 1000 min^{-1}, a scan rate of 1 mV·s^{-1}, and 25°C. a) and b) HER polarization curves versus Fc/Fc^{+} andAg/AgCl, respectively.

Anodic stability

Linear polarization experiments performed in WIS with increasing concentrations on a rotating glassy carbon electrode on oxidation as shown in Fig. S1. During positive polarization, the oxidation potential for water continuously shifts to increasing potentials with increasing concentration in agreement with previous work.[8] This increase in the stability of water was recently explained by molecular dynamics (MD) simulations that showed that the strong adsorption of TFSI at the positive electrode tends to push lithium ions and water molecules away from the electrode surface[8]. Our MD simulations for electrolyte concentrations of 3.5 and 20 m at carbon electrodes are in agreement with these findings (see the density profiles on Fig. S2a). Although for the more dilute 3.5 m electrolyte the density of TFSI is not sufficient to fully displace water, in other words to "dry" the first adsorbed layer, it is indeed the case in the WIS regime at 20 m concentration, as illustrated on the representative snapshots shown on Fig. S2b. Hence, high salt concentrations result in an additional thermodynamic barrier for water to reach the positive electrode surface and to get oxidized.

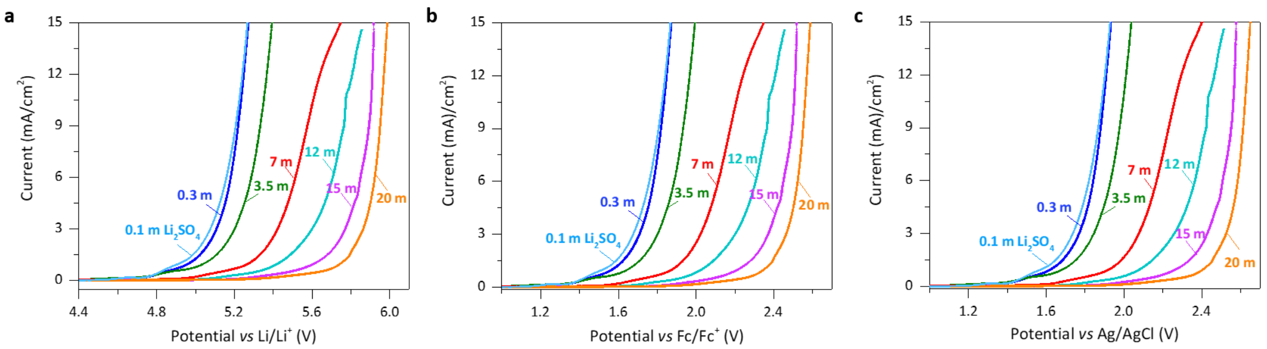

Figure S2. Electrochemical stability of LiTFSI-H_2O water-in-salt electrolytes. Linear polarization on a glassy carbon rotating disk electrode with different salt concentrations (molality). Platinum was used as counter electrode and Ag/AgCl as reference electrode. Curves were measured at 1000 min^{-1}, a scan rate of 1 mV·s^{-1}, and 25°C.´ a), b) and c). OER polarization curves versusLi/Li^{+}, Fc/Fc^{+} and Ag/AgCl respectively.

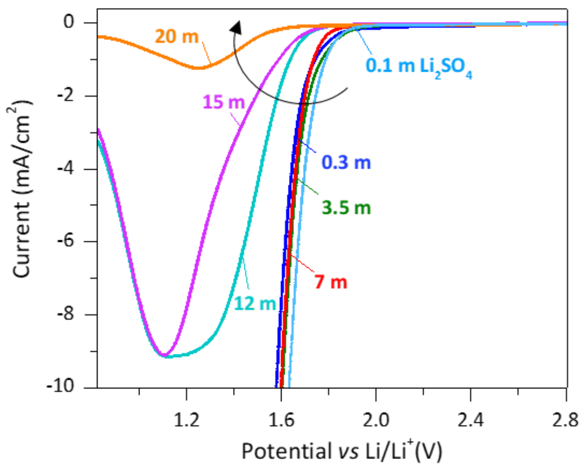

Figure S3. Kinetic contribution in WIS electrolyte: the current density of the reduction water decreases with the electrolyte concentration from 7 to 20 m, suggesting a kinetic effect of the reaction.

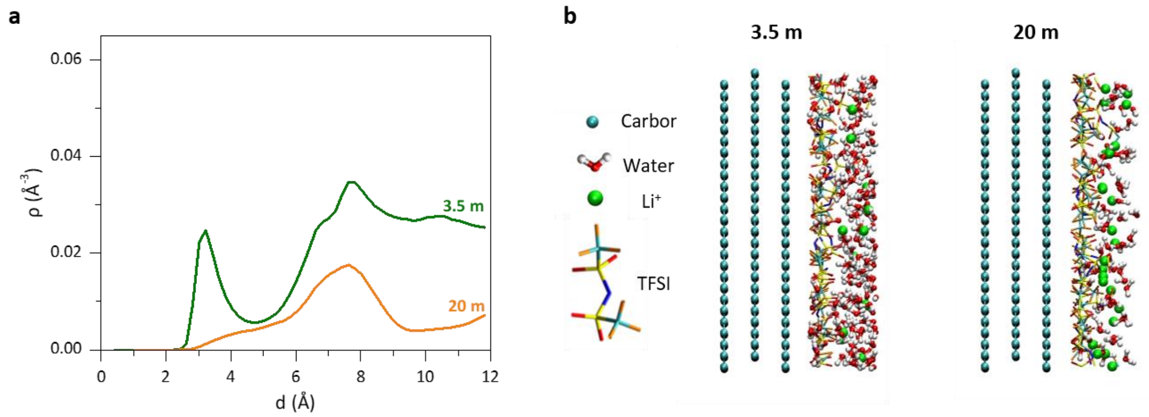

Figure S4. Interfacial water density at the positive electrode. a) Water density profiles from MD simulations at 3 V. b) Representative snapshots showing the presence/absence of water molecules in the first adsorbed layer with 3.5 and 20 m electrolytes, respectively.

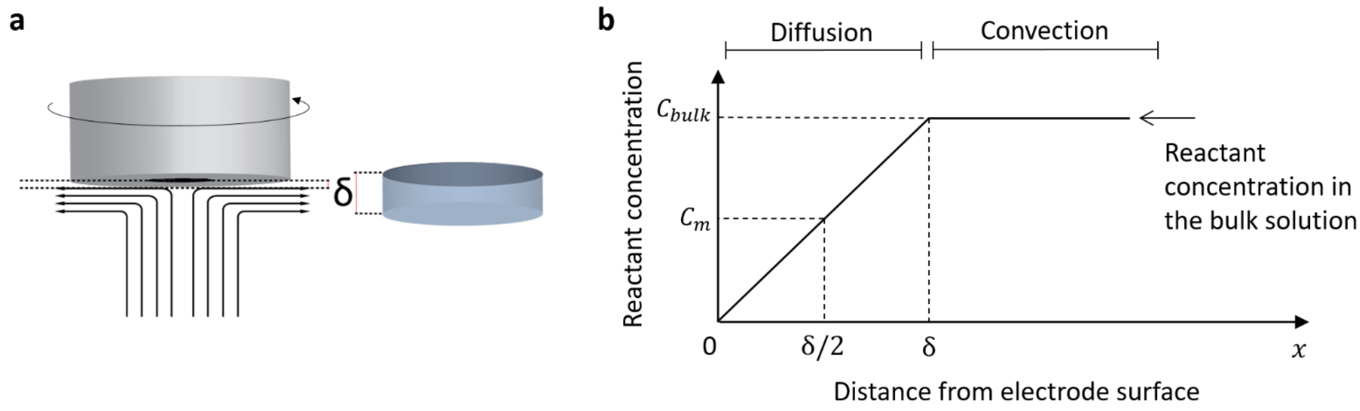

Figure S5. a) Schematic representation of the rotating disk electrode and the diffusion layer, **b)** representation of the diffusion layer profile model.

Water reduction at Mo_6S_8 electrodes

The results in Fig. 1 explain the reductive (cathodic) stability only on the basis of water reduction; direct TFSI reduction to form a passivating layer as suggested in previous reports on Chevrel phase Mo_6S_8 electrodes[8,9] appears not to be involved. To check whether the electrode material has an influence on the mechanism, we performed polarization measurements with porous electrodes made of the same Mo_6S_8. The results are compared to the ones on a porous carbon nanofiber (CNF) electrode in Fig. S6. The carbon electrode with 12 m solution shows analogously to Fig. 1a, a peaking higher voltage process and further reduction at lower voltage. The slight shift of the potentials is related to the different scan rate applied and to the porosity of the electrode surface. The Mo_6S_8 electrode shows a plateau with an onset at ~2.4 V with the 3.5, 12, and 20 m electrolytes, which was assigned to the reduction of Mo_6S_8[8,10]. As with graphite electrodes, there is a reduction onset at ~1.8 V. The current density of the second reduction decreases with the electrolyte concentration from 3.5 to 20 m, suggesting a kinetic effect of the reaction, which we finally attribute to water reduction. The same two reduction waves confirm that water reduction at two distinct potentials at carbon and Mo_6S_8 electrodes is governed in the same way by the chemical environment of water molecules.

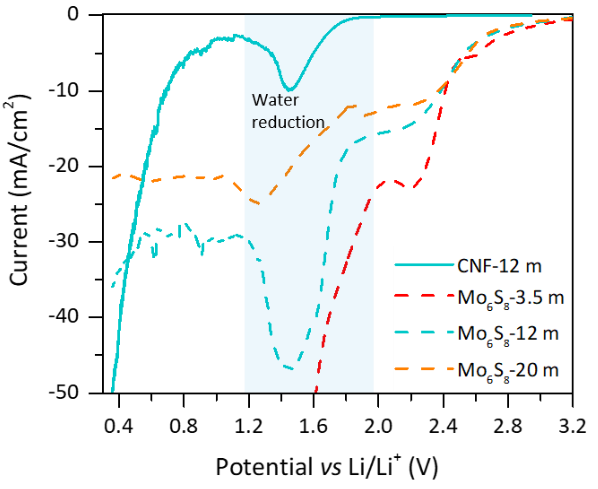

Figure S6. Linear polarization of carbon nanofiber (full line) and Mo_6S_8 (dashed lines) electrodes in the indicated WIS electrolytes at 5 mV·s^{-1}. Platinum disk was used as counter electrode and Ag/AgCl as reference electrode.

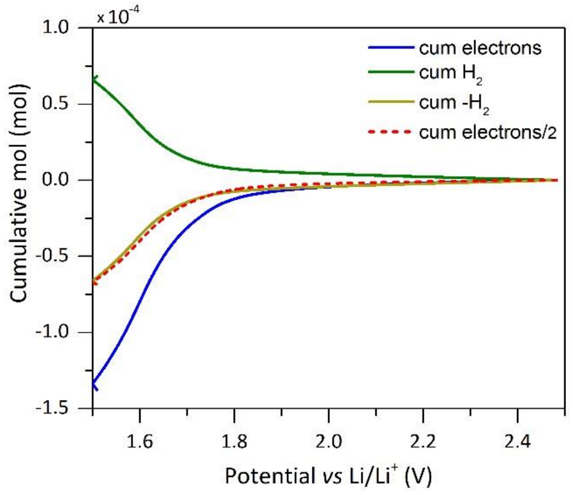

Figure S7. Cumulative moles of H_2 in comparison to the cumulative electrons and half the cumulative electrons with 20 molal LiTFSI in H_2O.

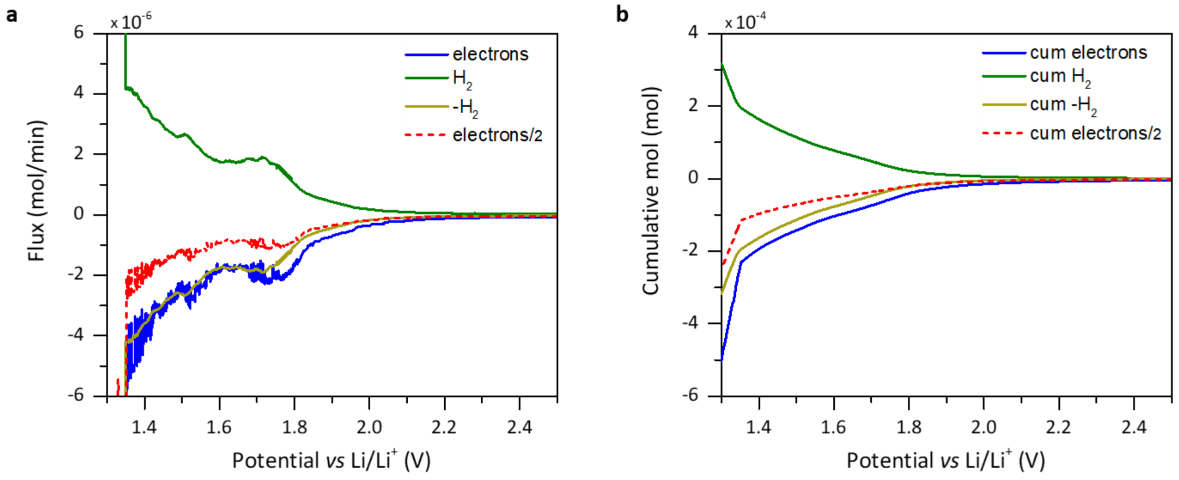

Figure S8. Operando online electrochemical mass spectrometry of a carbon black/PTFE composite electrode in 0.3 molal LiTFSI in H_2O as the electrolyte at a scan rate of 0.1 mV·s^{-1}. a). molar flux of H_2 in comparison to the electron flux and half the electron flux. b). cumulative moles of H_2 in comparison to the cumulative electrons and half the cumulative electrons.

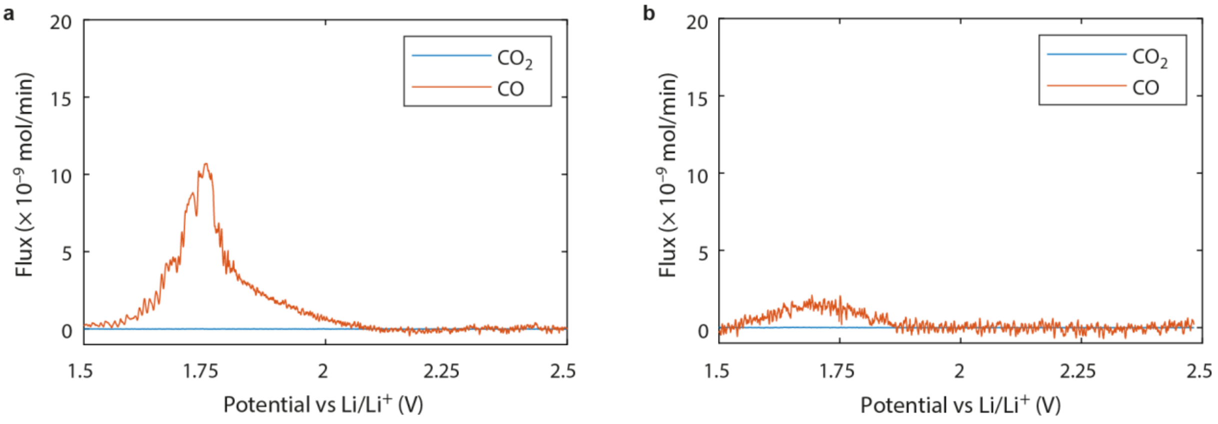

Figure S9. Molar flux of CO_2 and CO evolution during the online mass spectrometry measurements shown in Fig S8 for 0.3 m electrolyte. a) and in Fig. 3 for 20 m electrolyte.

Precipitation/dissolution mechanism at the interface

The laminar flow at a rotating disk electrode conveys a steady stream of material from the bulk solution to the electrode surface. The rotating structure acts as a pump, pulling the solution upward and then throwing it outward reaching a steady state rather quickly as demonstrated in Fig. S5a.[11,12]
According to the diffusion layer model, the electrolyte can be divided into two zones (Fig. S5b):
1. A first region close to the surface of the electrode with thickness δ, where it is assumed that there is a totally stagnant layer and thereby diffusion is the only mode of mass transport.
2. A second zone outside the first region where a strong convection occurs, and all species concentrations are inchanged during the redox mechanism.[11,12]

The thickness of the stationary diffusion layer at a rotating disc electrode is illustrated in Fig. S5a and is approximately described by the Levich equation:

$$\delta = 1.61 \cdot \nu^{0.166} \cdot D^{0.33} \cdot \omega^{-0.5} \qquad \text{(S1)}$$

where *v* is the kinematic viscosity, *D* the diffusion coefficient and *ω* the angular speed of the electrode.
In the first zone where there is a concentration gradient, the slope of the curve can be determined by using these following coordinates: concentration at δ distance (C_{bulk}, δ) and the average concentration at δ/2 (C_m, δ/2), figure S5b:

$$Slope = \frac{C_{bulk}-C_m}{\delta-\frac{\delta}{2}} = \frac{2(C_{bulk}-C_m)}{\delta} \qquad \text{(S2)}$$

The concentration change due to water reduction in the diffusion layer can be calculated by using Faraday's equation corresponding to the number of moles in the volume when a charge Q has been consumed

$$C_{cons} = \frac{N}{S\cdot\delta} = \frac{|Q|}{S\cdot\delta\cdot n\cdot F} \qquad \text{(S3)}$$

Here Q is the charge, *n* is the number of electrons exchanged during the reaction, *F* is the Faraday constant and *S* is the surface of the electrode. The average concentration is therefore the remaining water concentration.

$$C_m = C_{bulk} - C_{cons} = C_{bulk} - \frac{|Q|}{S\cdot\delta\cdot n\cdot F} \qquad \text{(S4)}$$

Thus, the slope can be expressed as a function of the charge, the diffusion layer and the concentration in the bulk:

$$Slope = \frac{C_{bulk}-C_m}{\delta-\frac{\delta}{2}} = \frac{2(C_{bulk}-(C_{bulk}-\frac{|Q|}{S\cdot\delta\cdot n\cdot F}))}{\delta} = \frac{2\cdot|Q|}{\delta^2\cdot n\cdot S\cdot F} \qquad \text{(S5)}$$

The concentration in the first zone ($C(x)$, at all value of x) can be expressed as function of the slope and the concentration at x=0:

$$C(x) = slope\cdot x + C_{x=0} \qquad \text{(S6)}$$

For any point in the diffusion layer with 0< x ≤ δ, the linear equation is valided. Hence, at x = δ the concentration gradient equation reads:

$$C_{x=\delta} = C_{bulk} = slope\ \delta + C_{x=0} = \frac{2\cdot|Q|}{\delta\cdot n\cdot S\cdot F} + C_{x=0} \qquad \text{(S7)}$$

Therefore, the concentration at the interface (x = 0) is given as follow:

$$C_{x=0} = C_{bulk} - \frac{2\cdot|Q|}{\delta\cdot n\cdot S\cdot F} \qquad \text{(S8)}$$

The concentration of LiTFSI varies linearly with the water concentration as shown in the Fig. S10. The linear equation was used to extrapolate the salt concentration at the interface. Note that, the determined concentration are apparent values because we have included concentration higher than the solubility limit. The results express instead the solubility of the salt at the interface. A concentration exceeding the solubility means that the interface is continuously renewed with water enabling the solubility of LiTFSI salt therefore the high concentration values.

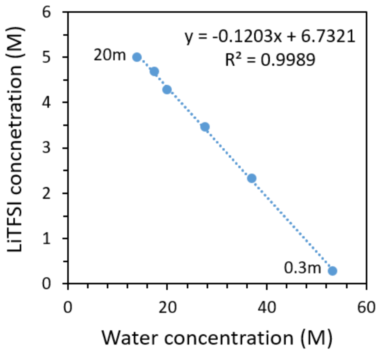

Figure S10. LiTFSI concentration as function of water concentration in different WIS solution form 0.3 to 20m (from 0.29 to 4.99M).

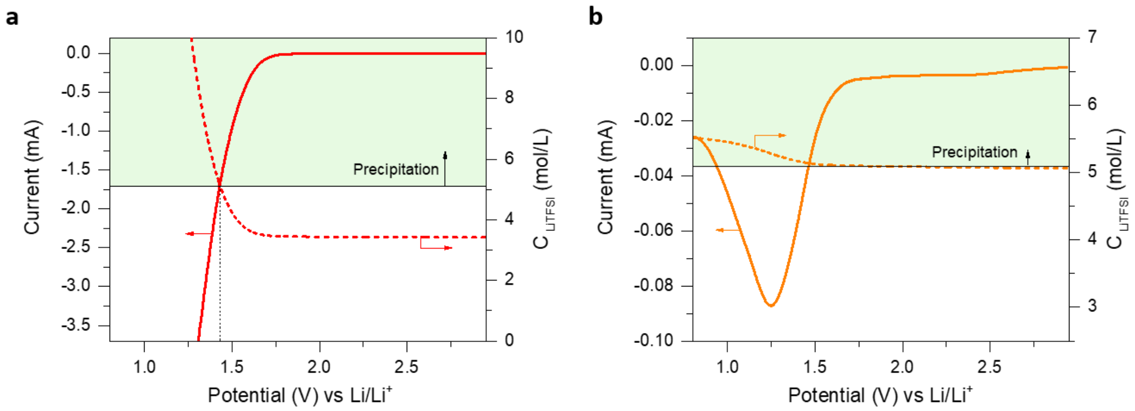

Figure S11. Salt concentration profile at the interface during a linear sweep voltammetry with 7 m (a) and 20 m (b) LiTFSI electrolytes. The solubility limit is ~5.1 M corresponding to 22-23 m and 25°C.

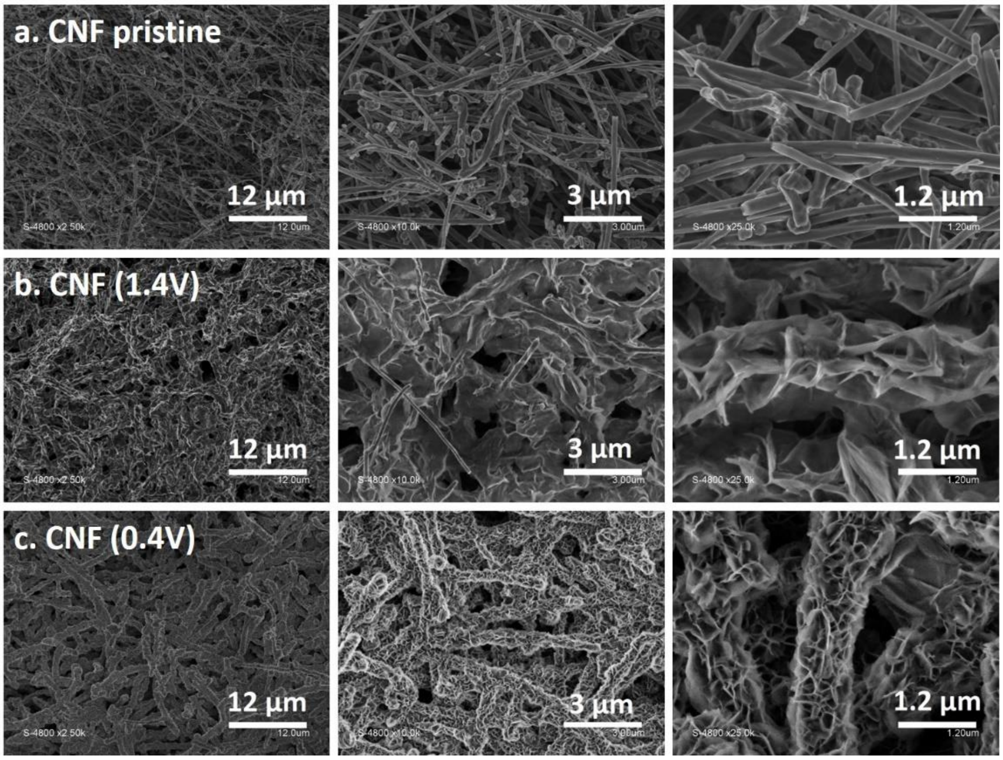

Figure S12. Scanning electron spectroscopy on carbon nanofibers in 12 molal WIS solution.

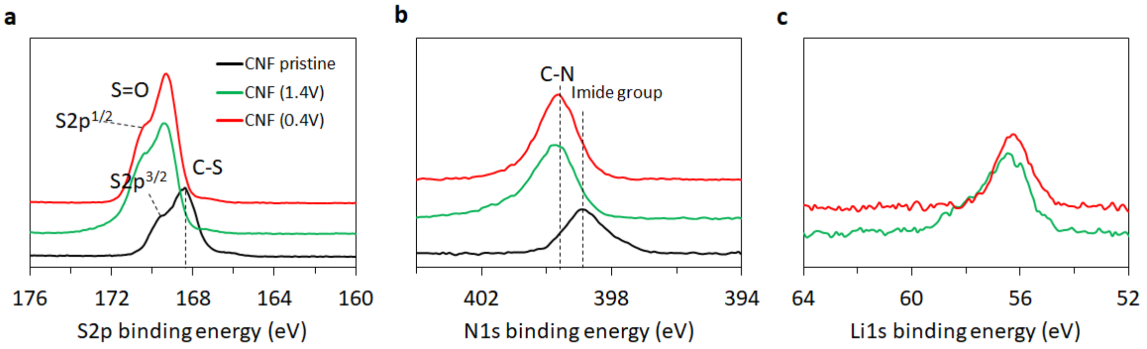

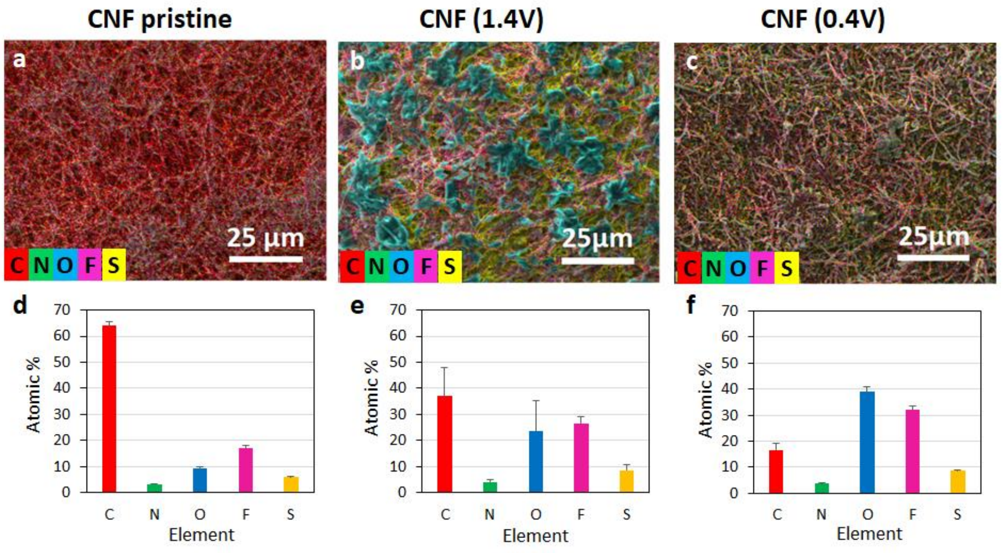

Figure S13. SEM and EDS analysis of carbon nanofibers in 12 m WIS solution. a to c, Spatial distribution of the different chemical elements at the electrode surface of pristine CNF (**d**), CNF polarized to 1.4 V (**e**) and CNF polarized to 0.4 V (**f**). **g to i**, EDS spectra of pristine CNF (**g**), CNF polarized to 1.4 V (**h**) and CNF polarized to 0.4 V (**i**).

Figure S14. X-ray photoelectron spectroscopy (XPS) spectrum of CNF nanofibers based electrode in 12 m WIS solution. Black, green and red curves correspond to the pristine CNF, CNF(1.4V) and CNF(0.4V) electrodes, respectively. (**a**), S2p (**b**) N1s and (**c**) Li1s.

Interface formation at Mo_6S_8

Mo_6S_8 electrodes were polarized to 2.5 V and 1.4 V vs. Li^+/Li, corresponding to the Mo_6S_8 reduction and water reduction potential, respectively. The electrodes were first polarized in 12 m electrolyte to various reducing potentials for 15 min to form a surface film and the pristine electrode was immersed overnight in 12m solution. The electrodes recovered and rinsed intensively with water to remove excess electrolyte or precipitated salt, then dried at 80 °C for 24 h.

In contrast to the CNF electrodes in Fig. S12, polarized Mo_6S_8 electrodes appear in the SEM virtually identical to the pristine electrode (Fig. S15). Nevertheless, EDS shows a drastically increased oxygen content at 2.5 V with the other atom ratios unchanged. At 1.4 V, the O fraction decreases slightly whilst leaving the other atom ratios unchanged (Fig. S16).

Figure S17 shows the C1s, O1s, F1s, S2p, N1s, and Li1s spectra of the Mo_6S_8 electrodes. The electrode polarized to 2.5 V shows peaks very similar to those of the pristine electrode without any deposit, except for C1s spectra showing the presence of CO_3 and CO_2 peaks, which can be explained by the adsorption of CO_2 during cell assembly. Therefore, polarization to the potential, where Mo_6S_8 is reduced does not result in a surface layer via electrolyte reduction. In contrast, largely the same decomposition products as on CNFs are observed at 1.4 V, with an additional peak characteristic for Li_2CO_3. The strong intensity of this latter peak may be assigned to the oxidation of the carbon counter electrode. This assertion contrasts with the reduction of trace CO_2 as suggested by Suo et al.[9] Our interpretation is supported by the absence of Li_2CO_3 at the CNF electrode, where the counter electrode was a platinum disk, excluding reductive processes at the negative electrode in the absence of CO_2 as the Li_2CO_3 source. To confirm that the positive electrode is the origin of CO_2, we polarized a symmetric cell with CNF electrodes as both cathode and anode. XRD of the negative electrode confirms the presence of Li_2CO_3 (Fig. S18) and thus carbon from the positive electrode to be the Li_2CO_3 source at the Mo_6S_8. Overall, carbon and Mo_6S_8 electrodes are covered with the same surface species when polarized to potentials that drive water reduction.

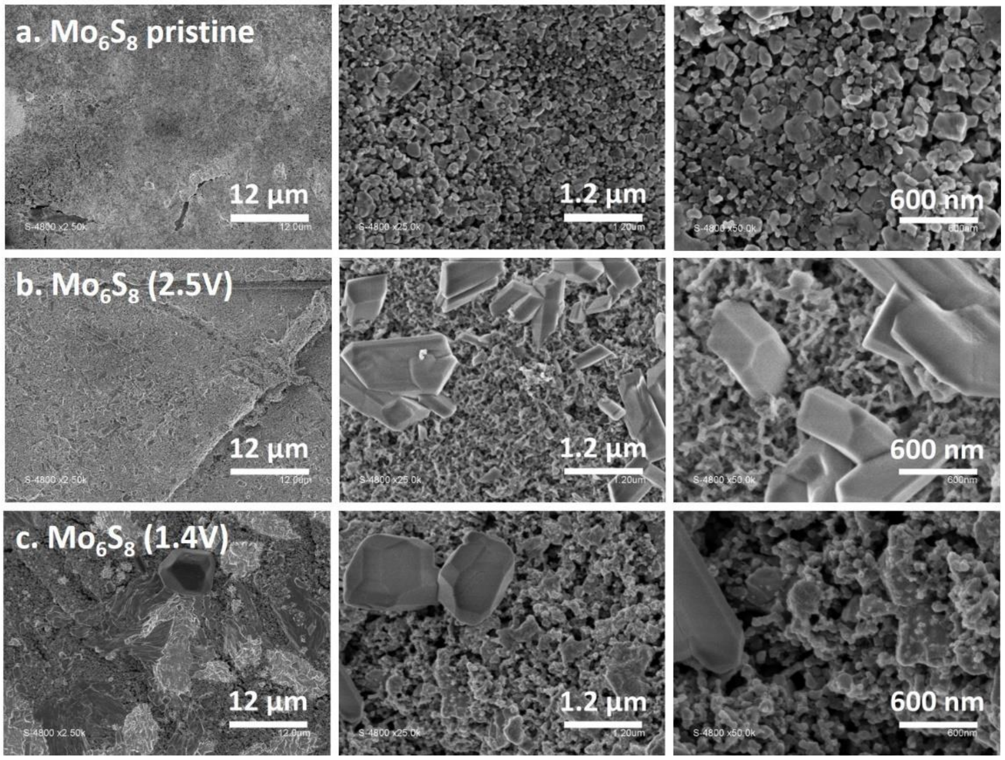

Figure S15. Scanning electron spectroscopy on Mo_6S_8 based electrode in 12 molal WIS solution.

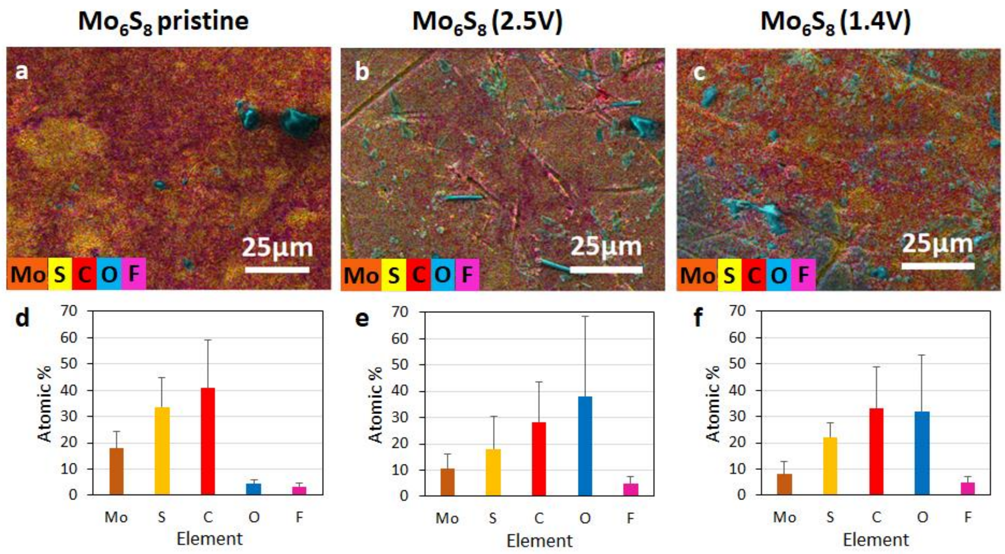

Figure S16. EDS analysis on Mo_6S_8 based electrode in 12 molal WIS solution. **a to c,** Spatial distribution of the different chemical elements at the electrode surface of pristine Mo_6S_8 (**d**), Mo_6S_8 polarized to 2.5 V (**e**) and Mo_6S_8 polarized to 1.4 V (**f**). **g to i**, EDS spectra of pristine Mo_6S_8 (**g**), Mo_6S_8 polarized to 2.5 V (**h**) and Mo_6S_8 polarized to 1.4 V (**i**).

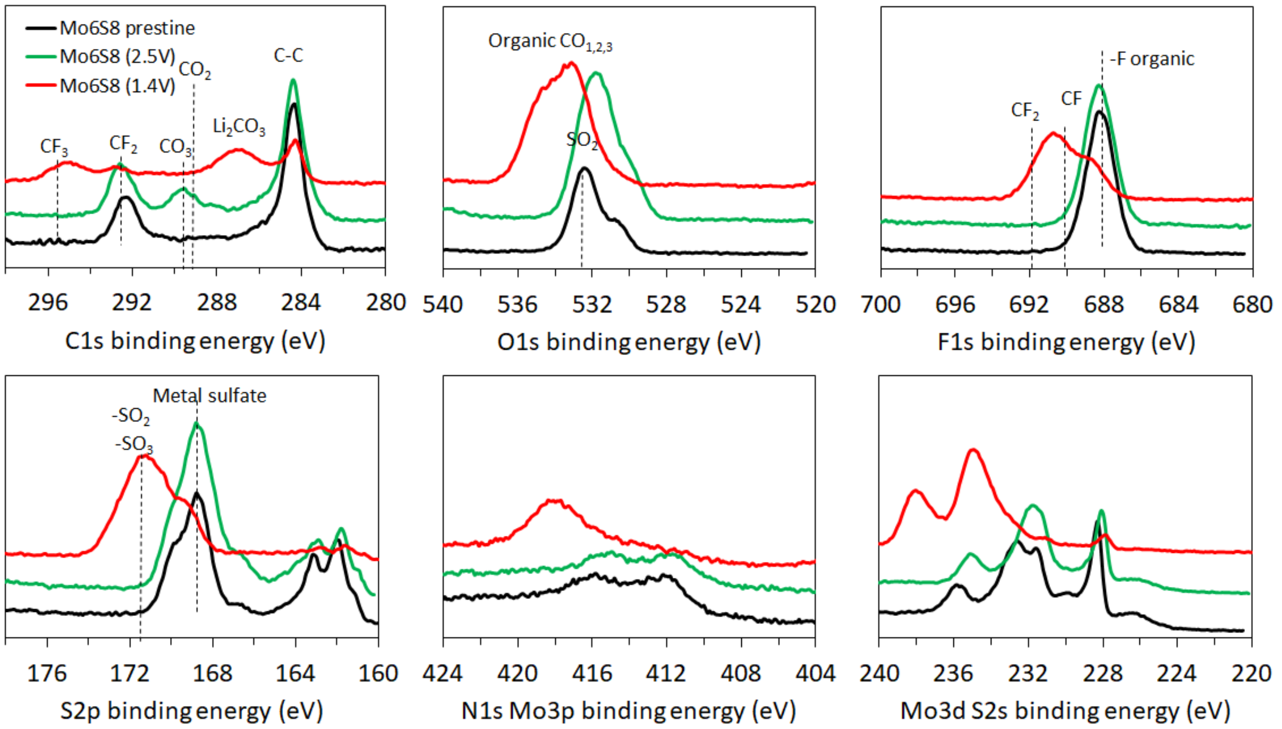

Figure S17. X-ray photoelectron spectroscopy (XPS) spectrum of Mo_6S_8 based electrode in 12 molal WIS solution. Black, green and red curves correspond to pristine Mo_6S_8, Mo_6S_8 (2.5V) and Mo_6S_8 (1.4V) electrodes, respectively.

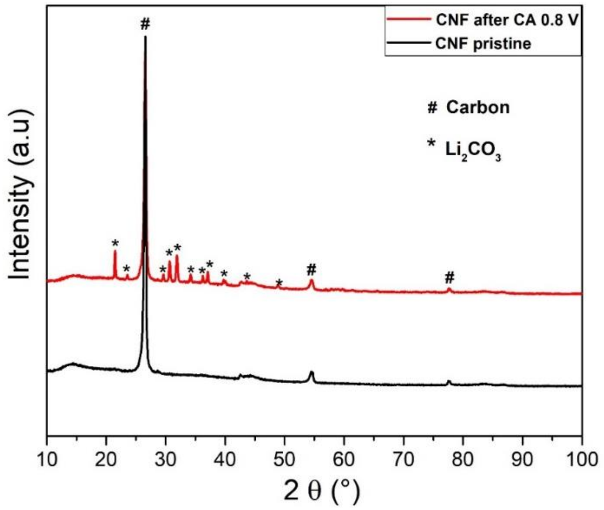

Figure S18. X-ray diffraction of pristine CNF electrode and after polarization at 0.8 V.

Supporting References

[1] M. J. Monteiro, F. F. C. Bazito, L. J. A. Siqueira, M. C. C. Ribeiro, R. M. Torresi, *J. Phys. Chem. B* **2008**, *112*, 2102–2109.

[2] H. J. C. Berendsen, J. R. Grigera, T. P. Straatsma, *J. Phys. Chem.* **1987**, *91*, 6269–6271.

[3] Z. Li, G. Jeanmairet, T. Méndez-Morales, B. Rotenberg, M. Salanne, *J. Phys. Chem. C* **2018**, *122*, 23917–23924.

[4] I.-C. Yeh, M. L. Berkowitz, *J. Chem. Phys.* **1999**, *111*, 3155.

[5] S. Plimpton, *J. Comput. Phys.* **1995**, *117*, 1–19.

[6] H. Yildirim, J. B. Haskins, C. W. Bauschlicher, J. W. Lawson, *J. Phys. Chem. C* **2017**, *121*, 28214–28234.

[7] V. Borgel, E. Markevich, D. Aurbach, G. Semrau, M. Schmidt, *J. Power Sources* **2009**, *189*, 331–336.

[8] L. Suo, O. Borodin, T. Gao, M. Olguin, J. Ho, X. Fan, C. Luo, C. Wang, K. Xu, *Science (80-.).* **2015**, *350*, 938–943.

[9] L. Suo, D. Oh, Y. Lin, Z. Zhuo, O. Borodin, T. Gao, F. Wang, A. Kushima, Z. Wang, H. C. Kim, et al., *J. Am. Chem. Soc.* **2017**, *139*, 18670–18680.

[10] L. Suo, F. Han, X. Fan, H. Liu, K. Xu, C. Wang, *J. Mater. Chem. A* **2016**, *4*, 6639–6644.

[11] A. J. Bard, L. R. Faulkner, *Electrochemical Methods : Fundamentals and Applications*, Wiley, **2001**.

[12] J. Nikolic, E. Expósito, J. Iniesta, J. González-Garcia, V. Montiel, *J. Chem. Educ.* **2000**, *77*, 1191.
